# Implementing Oxygen Therapy in Medical Wards—A Scoping Review to Understand Health Services Protocols and Procedures

**DOI:** 10.3390/jcm13185506

**Published:** 2024-09-18

**Authors:** Catherine Buchan, Yet Hong Khor, Toby Thomas, Natasha Smallwood

**Affiliations:** 1 Department of Respiratory Medicine, The Alfred Health, 55 Commercial Road, Melbourne, VIC 3004, Australia; natasha.smallwood@monash.edu; 2 Respiratory Research@Alfred, School of Translational Medicine, The Alfred Centre, Monash University, Melbourne, VIC 3004, Australia; yet.khor@monash.edu; 3 Department of Respiratory and Sleep Medicine, Austin Health, Heidelberg, VIC 3084, Australia; 4 Institute for Breathing and Sleep, Heidelberg, VIC 3084, Australia; 5 Melbourne Medical School, University of Melbourne, Grattan St and Royal Pde, Melbourne, VIC 3052, Australia; toby.thomas@swh.net.au

**Keywords:** oxygen therapy, local health policy and guidance, acute respiratory failure, ward-delivered

## Abstract

**Background/Objectives:** Conventional oxygen therapy (COT) is the cornerstone of management for hypoxaemia associated with acute respiratory failure (ARF) in wards. COT implementation guidance is provided in local health guidance documents (LHGDs). This study aimed to identify ward-delivered adult COT implementation LHGDs in Australian health services and assess their content and accuracy. **Methods:** A scoping review was conducted on 1 May 2022 and updated on 19 December 2023 to identify public health services COT LHGDs. Data were extracted and analysed regarding COT initiation, monitoring, maintenance and weaning, and management of clinical deterioration. **Results:** Thirty-seven included LHGDs, and eleven referenced the Australian COT guidelines. A definition in the LHGDs for hypoxaemia is that any oxygen saturation (SpO_2_) or arterial blood gas (ABG) is rare. None required ABG prior to COT initiation. Twenty-nine provided target SpO_2_ aims for initiation and maintenance. Fifteen did not specify the criteria for clinical review. Nine LHGDs provided guidance on weaning. **Conclusions:** There was considerable variation in the structure and content of COT LHGDs in Australian health services. Variations and limited guideline concordance of LHGDs may impact the quality and safety of health care. Considerations for future research include the development and implementation of standardised core LHGD recommendations for COT, as well as conducting a national oxygen audit to better measure and benchmark the safety and quality of care.

## 1. Introduction

Acute respiratory failure (ARF) is characterised by lung impairment leading to insufficient oxygenation (hypoxemia), ventilation (hypercapnia) or both. ARF is a leading cause of hospitalisation with high morbidity and mortality [1,2]. Optimising the management of ARF to improve survival is a key priority for clinicians. Non-invasive respiratory support therapy (NIRS) such as conventional oxygen therapy (COT), high-flow nasal oxygen (HFNO), and non-invasive ventilation (NIV) are important respiratory supports that may be delivered in medical wards to adults with ARF outside of intensive care units and emergency departments [3,4]. Of these supports, conventional oxygen therapy (COT) is the cornerstone of management for hypoxaemia associated with ARF.

COT can be delivered using low-flow nasal cannula or non-rebreather and venturi mask systems that achieve maximum flow rates of 10–15 litres per minute [5]. Often, COT is the preferred initial intervention for ARF over other forms of NIRS, given that it is low-cost, readily available, and non-invasive [6,7]. Nevertheless, appropriate use of COT requires careful prescription with specified target range aims of oxygen saturation for patients with and without hypercapnia, as well as clinical monitoring and review as recommended by professional societies such as the British Thoracic Society (BTS) [8,9]. These clinical practice guidelines recommend that healthcare organisations adopt an implementation approach for COT use, which includes education strategies and local audits to monitor clinical practice and patient outcomes [8,9,10].

However, implementation, adoption, and adherence to national and international clinical practice guidelines are the responsibility of healthcare organisations with well-recognised variations in care [11,12,13,14,15]. A key step in the implementation of COT is the development of local health guidance documents (LHGDs), including policies and procedural protocols informed by clinical practice guidelines, to provide technical information and clinical care standards to clinicians [16,17]. Heterogeneity in content and quality of LHGDs between health services may impact the quality and safety of patient care. Despite the importance of ward-based administration of COT, it is not known to what extent health services have specific LHGDs for COT, if these are informed by clinical practice guidelines, or the accuracy of the included information.

The aim of this study is to identify ward-delivered adult COT implementation LHGDs in Australian health services. Additionally, the study sought to assess their content and how they align with national and international clinical guidelines. We also aimed to develop a standardised minimum core set of information that clinicians require for ward-delivered adult COT implementation. We hypothesised that the structure and fundamental content of the healthcare organisations’ LHGDs would differ greatly.

## 2. Materials and Methods

A scoping review was conducted to detect and explore public health services’ LHGDs for adult ward-delivered COT implementation for patients with ARF in Australia. This scoping review protocol was registered on the Open Science Framework (https://osf.io/2ghyf) (accessed on 3 September 2024). The search was performed using the PROMPT database (https://prompt.org.au/) (accessed on 3 September 2024), which houses guidance documents online for 110 health services (public and private) in Victoria and New South Wales. This document repository enables participating health services to share LHGDs, aiming to support the provision of safe and high-quality clinical care among organisations, regardless of their available resources, including location and size.

On 1 May 2022, PROMPT was searched with the keywords ‘oxygen therapy’, which detected documents that included these words singularly or as a combination. The document search was updated on 19 December 2023. This search was complemented by incorporating LHGDs from healthcare organisations that did not use PROMPT during the study and were affiliated with the study team.

### 2.1. Inclusion and Exclusion Criteria

The inclusion criteria for identifying LHGDs were as follows, and documents meeting these criteria were incorporated into this review:

- Documents included local guidance of policy, procedure, protocol or guideline (or a combination);
- The title contained the term ‘oxygen therapy’ or similar;
- The content concentrated on adult ARF ward-delivered COT implementation.

COT LHGDs, which concentrated solely on COT use in paediatric or pregnant populations; oxygen delivery in the home, aged care, palliative care, intensive care, or emergency settings; COT use in ARF due to COVID-19; and HFNO only (without COT) were excluded. All LHGDs that met the inclusion criteria were reviewed, including those from healthcare services with more than one document.

### 2.2. Screening, Data Extraction, and Analysis

Two independent reviewers (T.T. and M.Z.) conducted title and full-text screening, with differences resolved with additional reviewers (N.S., Y.K., and C.B.) via discussion. Data were extracted using a specifically developed data extraction tool, including key components of (a) indications for COT, (b) COT initiation, monitoring, maintenance and weaning, and (c) overseeing clinical deterioration when using COT therapy and escalation of the care process. Two reviewers (C.B. and T.T.) independently extracted the data and cross-checked it. The full study team resolved any disagreements by discussion. Descriptive statistics using Microsoft Excel (v16.63.1) were used to analyse the data.

## 3. Results

**Figure 1:**
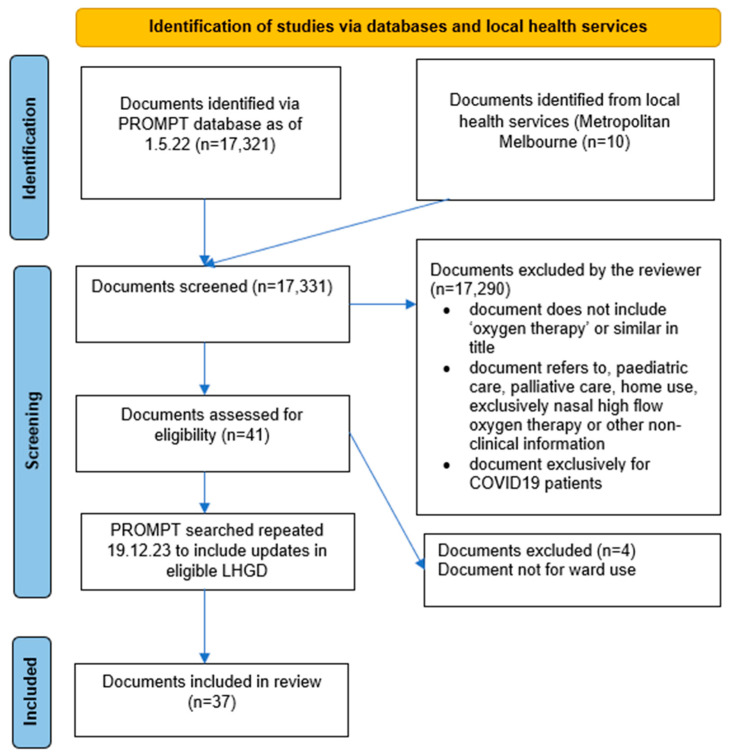
PRISMA diagram.

The initial search of the PROMPT database identified 17,321 documents, with ten additional documents added from Victorian health services not using PROMPT at the time of the study. After title screening, 41 documents underwent full-text review, with 4 documents being ineligible. The remaining 37 documents were included in the final review (Figure 1). No new documents were identified from the updated search in December 2023. However, 12 out of the 37 included documents from the initial search had been updated: 10 had new version identifications or logo amendments without content changes, and 2 had revisions with new information. The most up-to-date versions of LHGDs were included in this study.

### 3.1. Characteristics of Included Documents

The majority of the LHGDs were from rural health services (n = 21, 56.7%), followed by nine from regional (24.3%) and seven from metropolitan (18.9%) health services. Victorian health services LHGDs were the most represented (n = 36, 97.3%). The document types consisted of local guidelines (n = 17, 46.0%), combined policy and procedures (n = 6, 16.2%), policy (n = 8, 21.6%), and procedures (n = 6, 16.2%). Twelve (29.7%) LHGDs included HFNO recommendations, with others referring to separate HFNO LHGDs (n = 8, 21.6%). Six (16.2%) LHDGs had links to COT use in people with ARF associated with COVID-19. Nurses (n = 32, 86.5%) were the most often specified target audience of LHGDs, followed by doctors (n = 30, 81.1%), physiotherapists (n = 18, 48.6%) or all clinical staff involved in patient care (n = 18, 48.6%) (Supplementary Table S1).

The LHGDs infrequently referenced national and international acute oxygen therapy guidelines. Eleven documents (29.7%) referenced the 2015 Thoracic Society of Australia and New Zealand (TSANZ) Acute Oxygen Guidelines [18]; however, none referenced the revised 2021 TSANZ Acute Oxygen Position Statement [7] or the 2022 European Respiratory Society clinical practice guidelines [19]. The 2008 BTS Guideline for Emergency Oxygen Use in Adults was referred to in six documents (16.2%) [8], with the more recent 2017 BTS Guideline for Emergency Oxygen Use in Adults being referenced less often (n = 4, 10.8%) [9].

### 3.2. Indications for COT

All LHGDs provided a list of indications for COT, most commonly hypoxaemia (n = 37, 100%), followed by hypercapnic respiratory failure (n = 31, 83.8%), cyanosis (n = 11, 29.7%), and respiratory distress (n = 10, 27.0%) (Table 1). Most LHGDs (n = 23, 62.2%). did not provide a definition for hypoxaemia using one or the other of oxygen saturation (SpO_2_) or arterial blood gas (ABG) partial pressure of oxygen (PaO_2_). When definitions of hypoxaemia were specified, just over half (n = 22, 59.5%) stated SpO_2_ < 92% as a reason to consider COT initiation (Table 1). Pulse oximetry (n = 23, 62.2%) was more frequently recommended for assessment of the presence of hypoxaemia than ABG measurement (n = 13, 35.1%).

Less than half the documents included at least one contraindication for COT (n = 18, 48.6%), most commonly cigarette smoking (n = 18, 48.6%), followed by epistaxis (n = 4, 10.8%), base-of-skull fracture (n = 4, 10.8%), and carbon dioxide retention (n = 3, 8.1%). Most (n = 34, 91.9%) did not suggest consideration of goals of care prior to COT initiation. Nearly half of the documents recommended communication with the patients regarding oxygen use (n = 16, 43.2%), but none provided guidance on communication with non-English speakers.

### 3.3. COT Initiation and Maintenance

No LHGDs required ABG to be performed prior to initiation of COT (Table 2). One document (2.7%) stated that venous blood gases were not appropriate for the assessment of hypoxaemia. Most documents provided target SpO_2_ ranges for both initiation and ongoing treatments with COT. Recommendations for types and frequency of patient observations varied widely.

Medical staff were most often described as COT prescribers (n = 21, 56.7%), followed by nurses (n = 14, 37.8%) and physiotherapists (n = 4, 10.8%). The seniority of the medical nurses and physiotherapists who were able to initiate COT was not specified in 12 (32.4%), 22 (59.4%), and 33 (89.2%) of the LHGDs, respectively. No documents recommended the use of a COT prescription chart, yet two-thirds recommended that COT prescriptions include target SpO_2_ ranges (n = 25), followed by oxygen flow rate (n = 22, 59.4%), oxygen delivery device (n = 21, 56.7%), and frequency of observations (n = 15, 40.5%). Very few LHGDs specified the COT prescription location in the electronic medical record (EMR) (n = 3, 8.1%) or paper-based medical record (n = 3, 8.1%). Thirty-two documents (86.5%) described potential adverse events related to COT use, most commonly hypercapnia (n = 32, 86.5%), followed by cerebral hypoperfusion (n = 6, 16.2%), acute lung injury (n = 5, 13.5%), myocardial infarction (n = 4, 10.8%), and reabsorption atelectasis (n = 4, 10.8%). Guidance on the health professional clinical discipline (i.e., medical, nursing or physiotherapy) responsible for patient reassessment post-COT initiation, including adverse effects, clinical deterioration, or ongoing therapy required, was not specified in all LGHDs, including those that listed more than one clinical discipline such as nurses and doctors.

### 3.4. Escalation of Care

Maximum litres per minute of oxygen delivery and types of COT devices that could safely be implemented in the medical ward were not provided. Twenty-four (64.9%) documents specified COT devices that can achieve flow rates > 15 L per minute (Supplementary Table S1). Nearly half of the documents stated escalation to other NIRS, including HFNO (n = 15, 40.5%) and NIV (n = 18, 48.6%).

Twenty-two (59.4%) LHGDs contained at least one clinical parameter in COT users that required medical review, such as persistent hypoxaemia on COT despite increasing oxygen flow rate and no improvement in SpO_2_ while on COT. One document (2.7%) recommended escalation review for utilisation of COT for more than 4 days. Approximately half the documents (n = 19, 51.3%) specified a detection system for care escalation for patients deteriorating despite COT, most commonly the medical emergency criteria (n = 17, 45.9%) followed by the early warning score criteria (n = 2, 5.4%).

### 3.5. Weaning

Guidance for weaning patients from COT was rarely provided (n = 9, 24.3%). The majority of LHGDs (n = 34, 91.9%) did not specify COT device flow rate targets before commencing weaning. Twenty-nine documents (78.4%) did not provide guidance on which COT device to use (e.g., nasal cannulae or face mask) prior to COT cessation (Table 3). Less than a third of documents (n = 10) provided referral or ongoing clinical review advice for current or potential long-term oxygen therapy users (Supplementary Table S1).

Following the examination of the LHGDs, including the level of alignment with the current national and international COT recommendations [7,9], we developed a guidance template of essential information for ward-delivered COT implementation in adults with ARF (Table 4). This core information can be used to standardise COT implementation and improve the quality of care in the ward setting.

## 4. Discussion

This scoping review is the first to investigate LHGDs concerning the implementation of ward-delivered COT to adults with ARF. Key areas of insufficient information included inconsistent definitions of hypoxemia, variable target saturation ranges for COT, no oxygen prescription chart recommendations, limited guidance for COT initiation, maintenance and weaning, and inadequate information for detecting deteriorating patients. Importantly, guidance for treatment escalation to other NIRS (e.g., HFNO or NIV) was often lacking. The small number of LHGDs that included information on HFNO in the same document provided insufficient and varied information regarding initiation, safe monitoring and weaning of HFNO, and none referred to the European Respiratory Society HFNO guidelines [19]. Even fewer COT LHGDs alerted the reader that there were also separate relevant HFNO documents in their organisation. These discrepancies are noteworthy, as they may influence variations in patient care and result in clinician confusion, especially for those who work across multiple health services.

Most LHDGs did not provide a recommended SpO_2_ or ABG value to define hypoxemia; these parameters are crucial for assessing respiratory status and informing clinical decisions. The TSANZ Acute Oxygen Guidelines recommend initiating COT when people with ARF have SpO_2_ < 92% (or <88% for people at risk of hypercapnia) [7]. In addition, while the majority of LHGDs provided target SpO_2_ ranges for patients using COT, there was a lack of consistency with recommendations above and below the evidenced-based recommended SpO_2_ ranges. Target SpO_2_ ranges are key to minimising the harm associated with under-oxygenation [20,21] and over-oxygenation [22], yet concerningly, a study by Harper et al. found that patients with prescribed target SpO_2_ ranges spent significant time below and above these targets [23]. A meta-analysis by Chu et al. showed that oxygen administration above SpO_2_ 94–96% increases in-hospital mortality [24]. Furthermore, there is often a lack of appreciation that too much oxygen can cause harm and that adverse effects occur in those at risk of oxygen-induced hypercapnia [10,25]. Thus, it is vital that LHGDs recommend evidence-based target SpO_2_ ranges, including stating an upper limit for the range.

National and international acute COT guidelines have recommended written oxygen therapy prescriptions in the medical record and drug chart, including target oxygen saturation range, monitoring, and maintenance (e.g., titration) to achieve treatment aims [7,9]. Despite these recommendations, documentation of oxygen prescriptions is variable and often not guideline-concordant and may contribute to adverse events [26,27,28,29]. Anderson et al. study reports that over-oxygenation occurred in two-thirds of admissions in the setting of high levels of medical record oxygen prescription documentation but with inadequate documentation on prescription charts [30]. No LHDGs recommended the use of a COT prescription chart, and very few specified a documentation location in the medical record. There is no standardised COT prescription chart in Australia, and the national inpatient medication chart is not recommended for COT prescription, as the design elements do not effectively support the safe prescription, administration and monitoring of COT [31]. Notwithstanding, the development and use of a COT prescription chart, integrated into the EMR, is key to improved COT implementation, monitoring, and safe care.

While COT is used routinely in the ward setting for patients with ARF, some will fail COT and early deterioration detection and response is required to consider escalation to alternative NIRS such as HFNO or NIV, which may avoid transfer to higher acuity areas such as intensive care units (ICUs), and improve outcomes [32,33]. The physiological parameter most commonly recommended for monitoring patients on COT was SpO_2_, with respiratory rate and heart rate infrequently recommended and with considerable variability between LHGDs. However, respiratory rate is an important but under-utilised early indicator of clinical deterioration in medical wards, which can increase many hours before the oxygen saturations fall [34,35]. The frequency and timing of physiological observations were often lacking and also highly variable between LHGDs. The TSANZ and BTS Acute Oxygen Guidelines recommend using a physiological track and trigger system, such as an early warning score, to predict inpatient deterioration and identify at-risk patients [7,9]. Notably, there are many different early warning systems in use in different Australian hospitals [36]. In this study, half of the LHGDs specified a detection system, most commonly the modified early warning score used within the medical emergency team, for rapid care escalation for patients deteriorating despite COT [37,38]. No LHGDs in this study provided guidance as to the upper limit for ward-delivered COT flow, with one document recommending clinical review for those using COT for more than four days.

In this study, most LHGDs did not reference the national or international acute oxygen therapy clinical practice guidelines [7,9,19]. The TSANZ and BTS guidelines for acute oxygen therapy provide key evidenced-based recommendations regarding indications for COT use, monitoring required after initiation, and maintenance and weaning of COT [7,9]. Not drawing on or citing these key guidelines by LHGD authors may have occurred due to a lack of awareness or due to limited time and resources to review and update LHGDs as new guidelines are published [39]. A study by Alonso-Coello et al. found that half of the organisations had no standardised process for assessing whether a guideline is out of date, only a fifth would use external guidelines when updating or developing local guidelines, and two-thirds report that the current review and updating process lacks rigour [39]. Nevertheless, to improve clinical care provision and quality for patients using ward-delivered COT, it is crucial that LHGDs reflect national and international guidelines’ evidence-based recommendations.

The development and implementation of a standardised core LHGD template for ward-delivered COT is a mechanism that could be utilised to tackle LHGD inconsistencies. Such a template could then be adapted according to the local healthcare organisation’s context and service provision. In the United Kingdom, the BTS developed a national oxygen audit tool to support healthcare organisations in monitoring oxygen guideline implementation, and in hospitals that use electronic medical records (EMRs), it is possible to automate key guidance components to monitor oxygen use at the individual and hospital-wide levels [40,41]. This national program evolved to include all NIRS; however, in Australia, there is no similar national approach to audit COT implementation or examine any education provided to clinicians regarding COT.

While this study identified variations in recommendations for ward-delivered COT, it is unknown how the LHGDs are used by clinicians. Previous studies have shown under-utilisation of LHGDs and clinical practice guidelines due to a lack of awareness and preferential use of knowledge and clinical experience [29,42]. Additionally, writing a guideline for oxygen therapy alone will not change clinical practice without active measures (e.g., staff education—senior and junior; doctors, nurses, and physiotherapists, local champions, electronic medical record prompts and links to decision-aid tools, posters on the wall near oxygen source) to ensure effective implementation and behaviour change [29,42,43,44].

It is imperative that future research investigate LHGD awareness and use by clinicians in their daily practice and uncover the elements that hinder or aid their implementation. Additionally, monitoring health services with COT LHGD patient outcomes could illuminate the importance of the adoption of high-quality LHGDs for the safe delivery of COT.

### Strengths and Limitations

This is the first study to evaluate acute oxygen LHGDs to ascertain if core recommendations are consistent, based on current evidence, and provide sufficient information to support clinical decisions and application, to our knowledge. A scoping review approach with a clear protocol was utilised in this study. We used the PROMPT database, an LHGD-sharing portal utilised by over 100 health services in the largest states in Australia (by population of over 15 million) and across metropolitan, rural and regional areas [45]. A limitation of this study is that not all health services utilise the PROMPT portal; hence, the identified LHGDs in this study might not be representative of all LHGDs for COT in the ward setting.

## 5. Conclusions

Considerable variation in the composition and content of LHGDs for ward-delivered COT implementation in Australian public health services has been shown in this novel study. We identified significant variations in the guidance for the initiation, monitoring, and weaning of COT, as well as the mechanism for detecting patient deterioration when using COT. Such variations and limited guideline concordance of LHGDs may impact the quality and safety of health care. Considerations for future research include the development and implementation of standardised core LHGD recommendations for COT and conducting a national oxygen audit to better measure and benchmark the safety and quality of care.

## Figures and Tables

**Table 1:** Indications for COT.

	Count (n)	Frequency (%)
Indications for COT *		
-Hypoxemia	37	100
-Hypercapnic respiratory failure	31	87.8
-Cyanosis	11	29.7
-Respiratory distress	10	27.0
-Chest pain	4	10.8
-Cardiac arrest	4	10.8
-Trauma	4	10.8
-Haemorrhage and shock	4	10.8
-Sepsis	3	8.1
-Seizure	3	8.1
-Carbon monoxide poisoning	2	5.4
-Stroke and SpO_2_ < 95%	2	5.4
-Post-operative	1	2.7
SpO_2_ hypoxemia definition to trigger COT consideration		
-<92%	22	59.5
-<94%	9	24.3
-Definition not specified	6	16.2
-<88% ^ⱡ^	0	0
PaO_2_ (ABG) hypoxemia definition to trigger COT		
-Definition not specified	23	62.2
-<80 mmHg	14	37.8
Hypoxemia assessed with *		
-Pulse oximetry	23	62.2
-Arterial blood gas	13	35.1
-Not specified	2	5.4
Indications for performing an ABG		
-Not stated	20	54.1
-Hypoxaemia	13	35.1
-Risk of hypercapnia	10	27.0
-Reliable SpO_2_ not obtainable	5	13.5
SpO_2_ targets (lowest range level) once COT initiated		
-Not specified	8	21.6
-92%+	22	59.5
-94%+	7	18.9
SpO_2_ targets (highest range level) once COT initiated		
-Not specified	19	51.4
-96%+	11	29.7
-98%+	7	18.9

* Cumulative percentages may sum over 100 as categories are not mutually exclusive. ^ⱡ^ <88%: with chronic respiratory conditions, ABG: arterial blood gas, COT: conventional oxygen therapy, LPM: litres per minute, PaO_2_: partial pressure of oxygen, SpO_2_: pulse oximetry oxygen saturation, VBG: venous blood gas.

**Table 2:** Recommendations for implementation and monitoring—COT.

	At Initiation—COT		Post-Initiation—COT	
	Count (n)	Frequency (%)	Count (n)	Frequency (%)
ABG required	0	0	3	8.1
VBG used to assess need for COT	0	0	1	2.7
SpO_2_ target range	29	78.4	29	78.4
Additional SpO_2_ target range specified for patients identified as vulnerable to hypercapnic respiratory failure	25	67.6	25	67.6
COT device rate *			N/A	N/A
-Not specified	4	10.8		
-1–2 LPM	33	89.2		
-2–4 LPM	33	89.2		
->5 LPM	33	89.2		
Mode of delivery COT			N/A	N/A
-Not specified				
-Nasal cannulae	34	91.9		
-Face mask	34	91.9		
-Non-rebreather mask	26	70.3		
-HFNO	18	48.6		
-Venturi device	13	35.1		
-Oximiser (oxygen reservoir)	2	5.4		
Documentation of COT settings *	N/A	N/A		
-Target SpO_2_ range			25	67.5
-Device flow rate			22	59.4
-Delivery system			21	56.7
-Frequency of observations			15	40.5
Documentation of patient observations *	N/A	N/A		
0			36	97.3
-Respiratory rate			21	56.7
-‘Vital signs’ stated in the LHGD			13	35.1
-Heart rate			12	32.4
-Blood pressure			11	29.7
-Temperature			6	16.2
Time until first observations			N/A	N/A
-Clinical discretion	26	70.3		
-Not specified	9	24.3		
-0–1 h	1	2.7		
-2–4 h	0	0		
Observation frequency	N/A	N/A		
-Clinical discretion			27	73.0
-0–1 h			3	8.1
-2–4 h			2	5.4
-4+ h			5	13.5
Flow diagram provided for COT initiation	8	21.6	N/A	N/A

* Cumulative percentages may sum over 100 as categories are not mutually exclusive. ABG: arterial blood gas, COT: conventional oxygen therapy, LPM: litres per minute, N/A: not applicable, SpO_2_: pulse oximetry oxygen saturation, VBG: venous blood gas.

**Table 3:** Weaning recommendations for COT.

Characteristic	Count (n)	Frequency (%)
Maintain target SpO_2_ range when weaning		
Not specified	28	75.7
->92%	9	24.3
Patient observations to be documented *		
-Not specified	26	70.3
0	8	21.6
-Respiratory rate	4	10.8
-Heart rate	4	10.8
-‘Vital signs’	2	5.4
Flow rate targets before COT cessation **		
-Not specified	34	91.9
-1–4 LPM	3	8.1
Device to use prior to COT cessation **		
-Not specified	29	78.4
-Face mask and/or nasal cannulae	8	21.6

* Cumulative percentages may sum over 100 as categories are not mutually exclusive. ** Patients with no chronic obstructive pulmonary disease (see Supplementary Table S1). COT: conventional oxygen therapy, SpO_2_: pulse oximetry oxygen saturation.

**Table 4:** COT LHGD template.

Document purpose	To provide essential clinical guidance for ward-delivered implementation of COT (including core information for initiation, monitoring, escalation, and weaning) for ARF in adults
Target audience	Clinical staff that are educated in adult COT implementation, including doctors, nurses, and physiotherapists
Definitions	Hypoxaemia, as defined by ABG or SpO_2_ values, requires COT ABG PaO_2_ < 60 mmHg or SpO_2_ < 92% * * Target SpO_2_ may vary and is based on assessment and clinical judgement on individual patient circumstances such as hypercapnia, i.e., SpO_2_ < 88%
Indications	COT is the first strategy for patients with hypoxaemic ARF In patients not vulnerable to hypercapnia, i.e., SpO_2_ < 92% Patients identified as susceptible to hypercapnia (e.g., those with chronic obstructive pulmonary disease): SpO_2_ < 88% Prior to COT initiation, consideration and discussion with patients and carers of treatment preferences, including the goals of care, are essential and must be documented for all patients * Consideration for HFNO where local resources permit
Contraindications	Normoxia associated with dysfunctional breathing (i.e., breathlessness) Active reoccurring nasal bleeding Surgical procedures—nasal and sinus (i.e., recent) Trauma—maxillofacial (i.e., recent) Fractures—base of skull (including suspected)
Prescription	COT prescription chart * Preference integrated into the electronic medical record Target SpO_2_ range 92–96% 88–92% in the setting of hypercapnia Monitoring and maintenance (e.g., titration and weaning) of treatment aims
COT devices’ characteristics	Low-flow nasal cannulae - Used in mild hypoxaemia - Flow: 0.5–4 litres per minute - Well tolerated, tubing with soft prongs placed in the nares and secured behind the patient’s head or around their ears Simple mask - Used in mild to moderate hypoxaemia - Flow: 6–10 litres per minute - Flows under 6 litres per minute risk rebreathing carbon dioxide - Mask over face and nose (with exhalation holes) is secured with elastic strap Non-rebreather mask - Used in moderate to severe hypoxaemia - Flow: 15 litres per minute - Mask over face and nose with oxygen reservoir bag and one-way valve, secured with elastic strap Venturi mask - Used in those at risk of hypercapnic respiratory failure - Flow rate required to deliver a controlled percentage of oxygen will vary according to device used - Mask over face and nose, secured with elastic strap Consider humidification if flows above 2–4 litres per minute used for more than 24 h as medical oxygen has a drying effect on mucous membranes * Recommend including device pictures and instructional videos
Initiation	ABG measurement should be considered clinically indicated, particularly in patients suspected of hypercapnia, as there are considerable limitations with SpO_2_ and venous blood gases COT equipment preparation methods for initiation (local service to add detailed step-by-step information and pictures) COT device titration parameters (i.e., flow litres) to maintain specified target SpO_2_ range: Patients not vulnerable to hypercapnia SpO_2_ 92–96% Patients identified as susceptible to hypercapnia SpO_2_ 88–92% * * Consider known disorders that have an increased susceptibility to hypercapnic respiratory failure. Examples include but are not limited to severe kyphoscoliosis, severe chronic obstructive pulmonary disease, obesity hypoventilation syndrome, neuromuscular disorders and respiratory muscle weakness, or patients with a known previous episode of hypercapnia (i.e., acute or chronic) The clinical monitoring required, including the frequency needed, is based on individual patient assessment within the therapy targets. Documentation of the COT therapy response should include the following essential clinical physiological parameters -Respiratory and heart rate, oxygen saturation, and temperature Initiation assessment and monitoring to be conducted every 15 min in the first 60 min of therapy. This recommendation may be influenced by local service resources, including nurse-to-patient ratios, acuity of the patient, goals of care, and the clinical indication Assessment and maintenance to include COT *device used*, the titration (upwards or downwards) of the *device flow rate* (litres per minute) in order to achieve the target SpO_2_ ranges (as stated above)
Maintenance	Physiological parameter observation intervals will vary based on individual patient clinical requirements. As the patient improves and stabilises, the frequency of observations will lessen * (i.e., from 1 to 4 h) * Based on local service resources (as above) The change in observation frequency is to be documented alongside the essential clinical physiological parameters of the respiratory and heart rate, oxygen saturation and temperature, and the COT *device used* to include the *device flow rate* (litres per minute) in order to achieve the target SpO_2_ ranges (as stated above) Clinical consideration for an ABG should be based on the individual patient assessment, including the frequency required
Criteria for medical review	Physiological parameters changes, including increased - Respiratory rate - COT device flow rates (litres per minute) - Escalation of the COT device type used Increased oxygen flow to meet the SpO_2_ target range Maximal oxygen flow to meet the SpO_2_ target range Unable to meet the SpO_2_ target range Clinical uncertainty using COT Other: As directed by local service early warning system [add local hospital alert system]
Escalation pathway	Clinical deterioration may occur in adults with ARF using ward-delivered COT. As such, they required ongoing regular assessment and monitoring Local service early warning scores should be used to assist clinical decisions for deteriorating patients and to escalate care [Add in the local service clinical tool], i.e., modified early warning score (medical emergency team), including the referral mechanism to ICU (include how to contact the ICU junior and senior doctors, e.g., pager number) [Add in the local work instruction] local ward upper limits for ward-delivered COT, i.e., device flow or level of oxygen delivered
Weaning	Weaning is essential strategy to assess response to therapy When weaning, decrease the oxygen flow rate to maintain the SpO_2_ target range, e.g., flow of <1–2 litres per minute Perform a room air assessment when clinically indicated, including assessment of desaturation (SpO_2_) episode (i.e., the time to desaturate), including the documentation of the essential physiological parameters of the respiratory and heart rate, oxygen saturation, and temperature If patient is a known long-term COT user, consideration for ongoing COT requirement, i.e., do not wean and cease COT to room air. Provide a new revised COT prescription for rest, activity, and sleep as indicated Referral pathway for known or suspected long-term COT use (include contact method, e.g., respiratory registrar pager) On cessation of COT, transition the patient to room air. Individual patient assessment and monitoring is required. Recommend lessening the frequency of essential physiological parameters of the respiratory and heart rate, oxygen saturation, and temperature to four to six hourly (or as indicated)
COT ward-delivered palliation	Clinical discretion is required for COT utilisation to manage non-hypoxaemic dyspnoea at the very end of life in some patients. Patient preference should be considered (see above). COT is usually well tolerated as the patients can communicate while using most COT devices Monitoring and escalation criteria are not routinely used for COT users at the end of life. COT used in this setting is to provide symptom support. COT adjustment or cessation should be considered when the patient is comfortable
Associated documents	e.g., HFNO LHGD, NIV LHGD, escalation of care LHGD, COT manufacturer instructions
References	e.g., TSANZ Acute Oxygen Position Statement [7], BTS Guideline for oxygen [9], ERS HFNO clinical practice guidelines [19]
Authors and review	Clinical staff positions and professional groups Review date Planned next review date Planned monitoring aligned to updated national and international guidelines

ABG: arterial blood gas, ARF: acute respiratory failure, COT: conventional oxygen therapy, HFNO: high-flow nasal oxygen, ICU: intensive care unit, LHGD: local health guidance document, LPM: litres per minute, PaO_2_: partial pressure of oxygen, SpO_2_: pulse oximetry oxygen saturation, * noteworthy.

## Data Availability

The data used are not publicly available to maintain the anonymity of the health services that were included in the study.

## Supplementary Materials

The following supporting information can be downloaded at https://www.mdpi.com/article/10.3390/jcm13185506/s1, Table S1: Further characteristics of included LHGDs.
