# Supplementary material for: Implementing Oxygen Therapy in Medical Wards—A Scoping Review to Understand Health Services Protocols and Procedures
Source: J Clin Med. 2024 Sep 18;13(18):5506. doi: 10.3390/jcm13185506 (PMC11432628; doi:10.3390/jcm13185506)
Supplement: Supplementary file 1 [file jcm-13-05506-s001.zip › jcm-3168802-supplementary.pdf]

Supplementary Table S1: Further characteristics of included LHGD

	Count (n)	Frequency (%)
Document contributors*		
-Not specified	10	27.0
-Nursing staff	17	45.9
-Medical staff	8	21.6
-Physiotherapy staff	1	2.7
-Other	4	10.8
Year of [#]LHGD publication		
-Not specified	21	5.4
-2023	7	2.7
-2021-2022	7	18.9
-2019-2020	20	18.9
-2018 or prior		54.0
Date of last review		
-Not specified	3	8.1
-2023	8	21.6
-2021-2022	11	29.8
-2019-2020	6	16.2
-2018 or prior	9	24.3
Document length (pages)		
-0-5	16	43.2
-6-10	13	35.2
-11-15	6	16.
-16-20+	2	25.4
LHGD objective stated	37	100
Definitions provided	14	38.3
LHGD summary provided	13	35.1
COT system components specified	29	78.4
COT device flow rates provided		
1-4 LPM	32	86.5
5-10 LPM	32	86.5
11-14 LPM	28	76.7
>15 LPM	24	64.9
Not specified	5	13.5
Advantages of COT provided	0	0
Guidance for patient positioning provided	0	0
Nurse to patient ratio on [1]COT		
-Not specified	0	0
Maximum recommended oxygen on general ward -Not specified	0	0
Communicating with patient at time of oxygen initiation	16	43.2
Communication with family and carers requires oxygen	0	0

Guidance on communicating with English not first language	0	0
Weaning advice for LTOT<br>Current LTOT users<br>New LTOT referral	 1<br>9	 2.7<br>24.3

**Categories are not mutually exclusive and cumulative percentages may sum to over 100LHGD: Local health guidance document*, *COT: Conventional oxygen therapy, LTOT: Long term oxygen therapy, LPM: Litres per minute.*
